# Comprehensive Characterization of a Subfamily of Ca^2+^-Binding Proteins in Mouse and Human Retinal Neurons at Single-Cell Resolution

**DOI:** 10.1523/ENEURO.0145-24.2024

**Published:** 2024-09-20

**Authors:** Jun-Bin Liu, He-Lan Yuan, Gong Zhang, Jiang-Bin Ke

**Affiliations:** ^1^State Key Laboratory of Ophthalmology, Zhongshan Ophthalmic Center, Sun Yat-sen University, Guangdong Provincial Key Laboratory of Ophthalmology and Visual Science, Guangzhou 510060, China; ^2^Oujiang Laboratory (Zhejiang Lab for Regenerative Medicine, Vision and Brain Health), Wenzhou 325000, China; ^3^State Key Laboratory of Ophthalmology, Zhongshan Ophthalmic Center, Sun Yat-sen University, Guangdong Provincial Key Laboratory of Ophthalmology and Visual Science, Guangzhou 510060, China

**Keywords:** Ca^2+^-binding protein, coexpression, retinal neuron, splice variant

## Abstract

Ca^2+^-binding proteins (CaBPs; CaBP1–5) are a subfamily of neuronal Ca^2+^ sensors with high homology to calmodulin. Notably, CaBP4, which is exclusively expressed in rod and cone photoreceptors, is crucial for maintaining normal retinal functions. However, the functional roles of CaBP1, CaBP2, and CaBP5 in the retina remain elusive, primarily due to limited understanding of their expression patterns within inner retinal neurons. In this study, we conducted a comprehensive transcript analysis using single-cell RNA sequencing datasets to investigate the gene expression profiles of CaBPs in mouse and human retinal neurons. Our findings revealed notable similarities in the overall expression patterns of CaBPs across both species. Specifically, nearly all amacrine cell, ganglion cell, and horizontal cell types exclusively expressed CaBP1. In contrast, the majority of bipolar cell types, including rod bipolar (RB) cells, expressed distinct combinations of CaBP1, CaBP2, and CaBP5, rather than a single CaBP as previously hypothesized. Remarkably, mouse rods and human cones exclusively expressed CaBP4, whereas mouse cones and human rods coexpressed both CaBP4 and CaBP5. Our single-cell reverse transcription polymerase chain reaction analysis confirmed the coexpression CaBP1 and CaBP5 in individual RBs from mice of either sex. Additionally, all three splice variants of CaBP1, primarily L-CaBP1, were detected in mouse RBs. Taken together, our study offers a comprehensive overview of the distribution of CaBPs in mouse and human retinal neurons, providing valuable insights into their roles in visual functions.

## Significance Statement

Ca^2+^-binding proteins (CaBPs; CaBP1–5) are a subfamily of calmodulin-like Ca^2+^ sensors. We investigated the gene expression patterns of CaBPs in mouse and human retinal neurons and found notable similarities across these two species. Nearly all amacrine cell, ganglion cell, and horizontal cell types expressed CaBP1, while most bipolar cell types, including rod bipolar (RB) cells, expressed various combinations of CaBP1, CaBP2, and CaBP5. Mouse rods and human cones exclusively expressed CaBP4, whereas mouse cones and human rods coexpressed CaBP4 and CaBP5. Additionally, mouse RBs coexpressed CaBP1 and CaBP5, with all three CaBP1 splice variants being detected. Collectively, our study provides a comprehensive overview of CaBP distribution in mouse and human retinal neurons, crucial for understanding their roles in vision.

## Introduction

Ca^2+^-binding proteins (CaBPs) are EF-hand CaBPs with high sequence similarity to calmodulin (Haeseleer et al., 2000, 2002). These proteins interact with targets similar to those of calmodulin, such as voltage-gated Ca^2+^ (Ca_V_) channels but often exert opposing effects (Lee et al., 2002; Zhou et al., 2005; Findeisen and Minor, 2010; Oz et al., 2013; Hardie and Lee, 2016; Ames, 2021). CaBPs, including CaBP1, CaBP2, CaBP4, and CaBP5, are predominantly expressed in the retina (Menger et al., 1999; Haeseleer et al., 2000, 2004; Rieke et al., 2008; Sinha et al., 2016) and the cochlea (P. S. Yang et al., 2006; Cui et al., 2007; T. Yang et al., 2016, 2018; Picher et al., 2017). Furthermore, CaBP1, also known as caldendrin, is expressed in various brain regions, indicating its widespread functional significance (Seidenbecher et al., 1998; Haeseleer et al., 2000; Kim et al., 2014; Konietzny et al., 2022; Lopez et al., 2023, 2024).

It has been well established that CaBP4 is exclusively expressed in retinal photoreceptors and interacts with L-type Ca_V_1.4 channels to regulate Ca^2+^ influx and neurotransmitter release (Haeseleer et al., 2004; Shaltiel et al., 2012). Mutations in the *Cabp4* gene, which encodes CaBP4, lead to severe visual defects in mice and humans, albeit with distinct symptoms observed (Haeseleer et al., 2004; Zeitz et al., 2006; Littink et al., 2009; Aldahmesh et al., 2010; Bijveld et al., 2013; Khan et al., 2013). In the mouse retina, CaBP5 is expressed primarily in rod bipolar (RB) cells, Type 3 OFF cone bipolar cells (BCs), and Type 5 ON cone BCs (Haeseleer et al., 2000; Haverkamp et al., 2003a; Rieke et al., 2008). CaBP1 is expressed in Types 1 and 2 OFF cone BCs, as well as in some amacrine cells (ACs), while CaBP2 is found in Type 1 OFF cone BCs and Type 6 ON cone BCs (Sinha et al., 2016), raising the possibility that CaBP1 and CaBP2 might be coexpressed in Type 1 cone BCs. However, to date, there is no direct experimental evidence to support this hypothesis.

Mice lacking CaBP1, CaBP2, or CaBP5 have been generated to study the functions of these proteins in the retina. Despite their presence in certain BC types, genetic ablation of CaBP1, CaBP2, or CaBP5 surprisingly does not significantly alter either the overall retinal morphology or the b-wave amplitudes of electroretinograms (ERGs), the latter of which reflect the light-induced activities in BCs (Rieke et al., 2008; Sinha et al., 2016). Nevertheless, ganglion cell (GC) light responses are altered upon the removal of each CaBP, indicating that CaBP1, CaBP2, and CaBP5 are involved in regulating signal transmission within the inner retina.

The incomplete understanding of the distribution of CaBP1, CaBP2, and CaBP5 within inner retinal neurons is a significant obstacle to fully elucidating their roles in retinal functions. Our work was motivated by a series of recent studies that have leveraged single-cell RNA sequencing (scRNA-seq) to achieve molecular classification of retinal cells in both mice and humans. As a result, transcriptomic profiles of various molecularly distinct retinal cell types are now accessible through the European Genome-phenome Archive (EGA accession number, EGAS00001004561) and Gene Expression Omnibus Database (GEO accession numbers, GSE63473, GSE81905, GSE149715, GSE137400, and GSE148077; Macosko et al., 2015; Shekhar et al., 2016; Tran et al., 2019; Cowan et al., 2020; Yan et al., 2020a,b). We conducted a reanalysis of these datasets, focusing specifically on the transcripts of CaBP-related genes. We found that the overall expression patterns of CaBPs in the retinas of mice and humans were very similar. Notably, almost all AC, GC, and horizontal cell (HC) types exclusively expressed CaBP1. In contrast, most BC types expressed different combinations of CaBP1, CaBP2, and CaBP5. The coexpression of CaBP1 and CaBP5 in mouse RBs was confirmed by single-cell reverse transcription polymerase chain reaction (scRT-PCR). The detection of three CaBP1 splice variants in RBs, then, indicated that the expression patterns of CaBPs in BCs were far more complex than expected.

## Materials and Methods

### Animals

All animal procedures were performed in accordance with the Oujiang Laboratory and Sun Yat-sen University animal care committees’ regulations. The wild-type C57BL/6J mice of either sex, aged postnatal day (P)17 and P42–70, were used for scRT-PCR analysis. We made every effort to minimize the number of animals used and their discomfort.

### Analysis of scRNA-seq datasets

The existing scRNA-seq datasets, encompassing GEO accession numbers GSE63473 (featuring transcriptomes of photoreceptors and HCs derived from P14 mice), GSE81905 (featuring transcriptomes of BCs derived from P17 mice), GSE137400 (featuring transcriptomes of ACs derived from P56 mice), and GSE149715 (featuring transcriptomes of GCs derived from P19 mice), were reanalyzed to examine the gene transcription patterns of CaBPs in the mouse retina (Macosko et al., 2015; Shekhar et al., 2016; Tran et al., 2019; Yan et al., 2020a). Demultiplexing and alignment of sequencing reads were performed, and individual digital expression matrices were generated for subsequent clustering analysis. Each gene expression matrix underwent quality control and normalization process as described previously (Macosko et al., 2015; Shekhar et al., 2016; Tran et al., 2019; Yan et al., 2020a; Tang et al., 2022; Zhang et al., 2022). Batch corrections were also applied for each gene expression matrix. Randomized principal component analysis was then used to reduce the dimensionality of the data. Using the Louvain–Jaccard algorithm (Blondel et al., 2008; Levine et al., 2015), transcriptionally resemble cells were partitioned into molecularly distinct clusters. Finally, gene expression patterns of CaBPs in different types of retinal neurons (i.e., different clusters) were shown as dotplots: the dot size represented the percentage of expressing cells in the cluster in which expression of a specific gene was detected, and the color intensity represented the average expression level of a gene in expressing cells.

Two existing scRNA-seq datasets [European Genome-phenome Archive accession number, EGAS00001004561 (dataset derived from four donors aged between 50 and 80 years); GEO accession number, GSE148077 (dataset derived from seven donors aged between 53 and 78 years)], which contain transcriptomes of different types of human retinal neurons, were reanalyzed to determine the gene expression patterns of CaBPs in the human retina (Cowan et al., 2020; Yan et al., 2020b).

### Single-cell RT-PCR analysis

To investigate the expression profiles of CaBPs and CaBP1 splice variants in individual RBs, we harvested the cytoplasmic contents of RBs from both P17 and adult wild-type mice using patch pipettes. Retinas were isolated from enucleated eyes in oxygenated (95% O_2_/5% CO_2_) Ames’ medium (Sigma-Aldrich) and then embedded in low-melting temperature agarose (Sigma-Aldrich type VIIA; 2% in a HEPES-buffered saline). Retinal slices (200 µm thickness) were cut on a vibratome (Leica VT1200s) and stored in oxygenated Ames’ medium at room temperature until use.

Patch pipettes (6–8 MΩ) were autoclaved to inactivate RNases and then filled with the pipette solution containing the following (in mM): 110 K-gluconate, 5 NaCl, 10 HEPES, 1 BAPTA, 8 PO_4_-creatine, 4 ATP-Mg, 0.4 GTP-Na_3_. The pH value was adjusted with KOH to 7.2. Recombinant ribonuclease inhibitor (catalog #2313A, Clontech) was included in the pipette solution to prevent mRNAs from degradation. To visualize the cell morphology of RBs on freshly cut retinal slices, Alexa Fluor 488 dye was also added into the pipette solution. Once the whole-cell patch–clamp configuration was established, cytoplasmic contents of RBs were harvested into the pipettes by gently applying negative pressure and then transferred into PCR tubes. PCR tubes were immediately frozen at −80°C until use. The pipette solution without any cell contents was used as a negative control.

Reverse transcription and sequence-specific amplification of mRNAs in the harvested cell contents were performed using the Single Cell Sequence Specific Amplification Kit (catalog #P621-A, Vazyme Biotech). Specific primer pairs (Table 1) were added into the reaction mixture with a final concentration of 0.1 µM for each primer. Reverse transcription was performed at 50°C for 60 min, followed by reverse transcriptase inactivation and Taq polymerase activation by heating to 95°C for 3 min on C1000 Touch Thermal Cycler (Bio-Rad Laboratories). After going through 20 cycles of sequence-specific amplification by denaturing at 95°C for 15 s, cDNAs were annealed and elongated at 60°C for 15 min. The preamplified products were then diluted and reamplified by another round of PCR with specific primer pairs for CaBP1, CaBP5, or CaBP1 splice variants (Table 1) using Phanta Max Super-Fidelity DNA Polymerase (catalog #P505-d1/d2/d3, Vazyme Biotech). The reamplified products were electrophoresed through 3% agarose gel, stained with SYBR Safe DNA Gel Stain (catalog #S33102, Invitrogen), and imaged under UV light exposure. Images were edited using the Prism 6 (GraphPad) and Photoshop (Adobe Systems) software.

**Table 1.** Primer sequences for scRT-PCR analysis

Gene		Forward primer (5′—3′)	Reverse primer (5′—3′)
*Cabp1*		TGACAGATCGTTACGACCAGA	GCCACCCAGGTTCATGTTG
*Cabp5*		ATGAGAACGATGGGTTACATGC	TCTCTGCAAGCAATTTGGGGG
*Prkca*		GCAGGGTGTTTGGTCATAAGT	GGGAAGTCTGTAGATTGGTGG
CaBP1 splice variants	GenBank ID number	Forward primer (5′—3′)	Reverse primer (5′—3′)
S-CaBP1/L-CaBP1	AF169153.1	TCTTTGTCAAATTCTCGGAAGG	GTCAAGTCGCCACTAAGAAACC
L-CaBP1	AF169152.1	TACATGGCGGTGCAGACAAG	TTCGGAGAAAAATGCAGGCG
Caldendrin	KJ364651.1	CGGCAGTTGATGTAACCATCCTTG	CCATGCTCAGCTCCGCCTTCG

CaBP1, Ca^2+^-binding protein 1; S-CaBP1, the short form of CaBP1; L-CaBP1, the long form of CaBP1.

## Results

### Analysis of scRNA-seq datasets reveals similar expression patterns of CaBPs in mouse retinal ACs, GCs, and HCs

**Figure 1.**
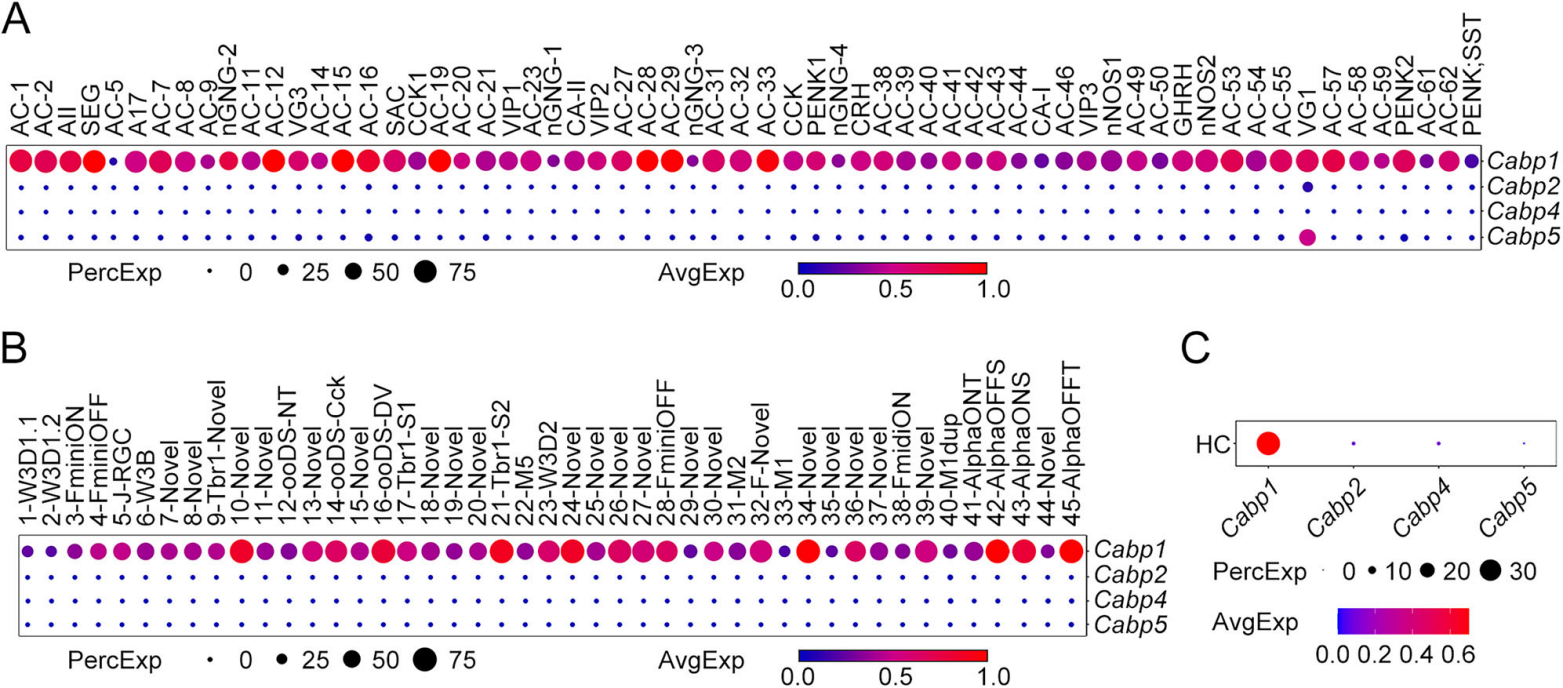
Similar expression patterns of CaBPs in mouse retinal ACs, GCs, and HCs. ScRNA-seq analysis reveals the gene expression patterns of CaBPs in 63 molecularly defined AC types (***A***), 46 GC types (***B***), and the sole HC type (***C***). The dot size represents the percentage of cells expressing a specific gene within each cluster (PercExp). The color indicates the average expression level of a gene in those expressing cells (AvgExp). CaBPs, Ca^2+^-binding proteins; AC, amacrine cell; GC, ganglion cell; HC, horizontal cell.

We began by analyzing the expression patterns of CaBP1, CaBP2, CaBP4, and CaBP5 in different types of mouse retinal neurons using several accessible scRNA-seq datasets (GEO accession numbers, GSE63473, GSE81905, GSE149715, and GSE137400; Macosko et al., 2015; Shekhar et al., 2016; Tran et al., 2019; Yan et al., 2020a). The dotplots within the figures offer a comprehensive visualization of gene expression patterns, with the dot size representing the percentage of expressing cells within a specific cell type and color intensity reflecting the average expression level of a gene among those expressing cells. In general, we found that mouse retinal ACs, GCs, and HCs shared very similar expression patterns of CaBPs (Fig. 1).

In the mouse retina, ACs have been classified into 63 distinct types by scRNA-seq in a recent study (GEO accession number, GSE149715; Yan et al., 2020a). Our analysis revealed that the vast majority of these molecularly defined AC types exclusively expressed CaBP1 transcripts (Fig. 1*A*). Specifically, we found that, with the exception of a GABAergic AC type designated as VG1, which exhibited high levels of both CaBP1 and CaBP5 transcripts (encoded by the *Cabp1* and *Cabp5* genes, respectively) and possibly a moderate expression of CaBP2 transcripts (encoded by the *Cabp2* gene), CaBP1 transcripts were the sole CaBP expressed in nearly all AC types (Fig. 1*A*). These findings are consistent with previous reports on CaBP1/caldendrin expression in rat and mouse ACs, primarily detected through in situ hybridization and immunohistochemistry (Menger et al., 1999; Haeseleer et al., 2000; Haverkamp et al., 2003a; Sinha et al., 2016).

The expression of CaBPs in retinal GCs has rarely been reported so far. Previous observations of CaBP1/caldendrin staining in the GC layer were often attributed to displaced ACs (Menger et al., 1999; Haverkamp et al., 2003a; Sinha et al., 2016). To delve deeper into the expression profiles of CaBPs in GCs, we analyzed another existing scRNA-seq dataset (GEO accession number, GSE137400) that encompasses single-cell transcriptomes of 46 molecularly defined mouse GCs (Tran et al., 2019). Remarkably, our analysis revealed that CaBP1 transcripts were exclusively expressed at appreciable levels across all GC types (Fig. 1*B*).

The expression of CaBP1/caldendrin in retinal HCs appears to be species-dependent, as reported in previous studies. For instance, HCs in the carp retina express caldendrin, whereas those in the rat retina do not (Menger et al., 1999; Schultz et al., 2004). Given this variability, we reanalyzed an existing scRNA-seq dataset (GEO accession number, GSE63473) which contains single-cell transcriptomes of mouse HCs (Macosko et al., 2015). Notably, we found that the sole HC type in the mouse retina exclusively expressed CaBP1 transcripts (Fig. 1*C*).

Taken together, these results demonstrated that nearly all AC, GC, and HC types in the mouse retina exclusively express CaBP1.

### Unique expression patterns of CaBPs in mouse retinal BCs

Given our observations of CaBP1 expression in ACs, GCs, and HCs (above), as well as previous reports of CaBP4 expression in photoreceptors, it is reasonable to expect relatively simple expression patterns of CaBPs in mouse retinal BCs. To further investigate this, we reanalyzed another existing scRNA-seq dataset (GEO accession number, GSE81905) that contains single-cell transcriptomes of 15 molecularly defined BC types in the mouse retina (Shekhar et al., 2016), with particular attention on the transcripts of CaBP-related genes.

**Figure 2.**
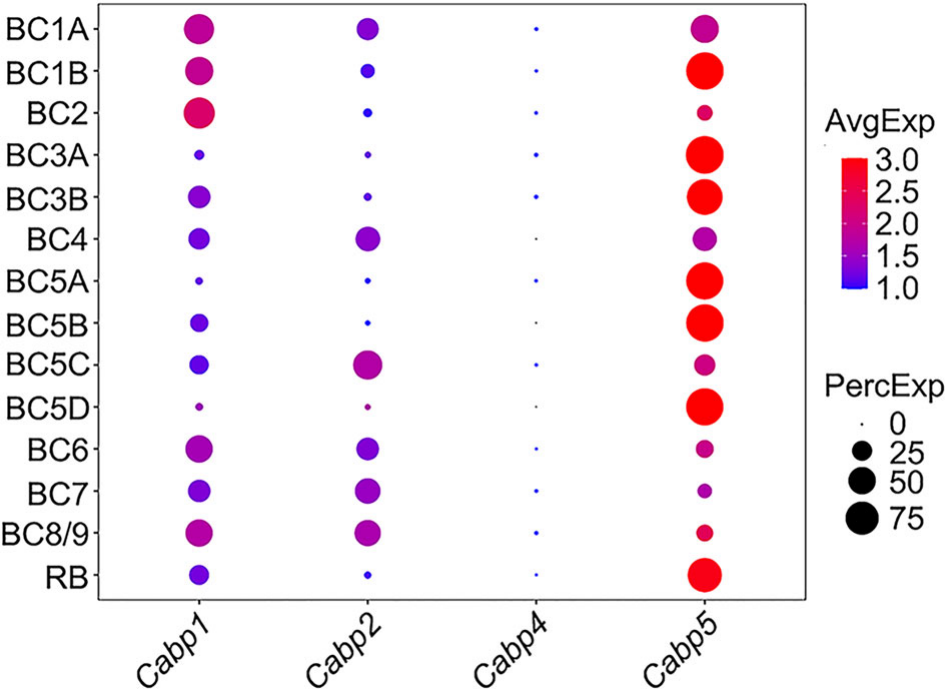
Unique expression patterns of CaBPs in mouse retinal bipolar cells. ScRNA-seq analysis reveals that distinct combinations of CaBP1, CaBP2, and CaBP5 are expressed in most BC types, including RBs. The dot size represents the percentage of cells expressing a specific gene within each cluster (PercExp). The color indicates the average expression level of a gene in those expressing cells (AvgExp). CaBPs, Ca^2+^-binding proteins; BC, bipolar cell; RB, rod bipolar cell.

The gene expression patterns of CaBPs in mouse BCs, however, were much more intricate than expected. CaBP5 transcripts were expressed at high levels in RBs, Types 3A and 3B OFF cone BCs, and Types 5A, 5B, and 5D ON cone BCs (Fig. 2), consistent with previous observations (Haeseleer et al., 2000; Haverkamp et al., 2003a; Rieke et al., 2008). Nevertheless, mRNAs encoding CaBP5 were also detected at appreciable levels in other BC types such as Types 1A, 1B, and 4 BCs (Fig. 2), indicating a broader expression pattern than previously reported. Similarly, CaBP1 transcripts were not limited to Types 1A, 1B, and 2 OFF cone BCs as reported previously (Sinha et al., 2016). Indeed, we found them expressed in many other BC types, including the RB (Fig. 2). Moreover, CaBP2 transcripts were detected at reasonable levels in Types 4, 5C, 7, and 8/9 cone BCs in addition to previously reported Types 1A and 6 cone BCs (Fig. 2; Sinha et al., 2016). In contrast, mRNAs encoding CaBP4 were generally detected at extremely low levels in all BC types (Fig. 2). Collectively, different combinations of CaBP1, CaBP2, and CaBP5 transcripts were observed in most BC types. This finding is quite striking given the general assumption that each BC type expresses only a single CaBP.

**Figure 3.**
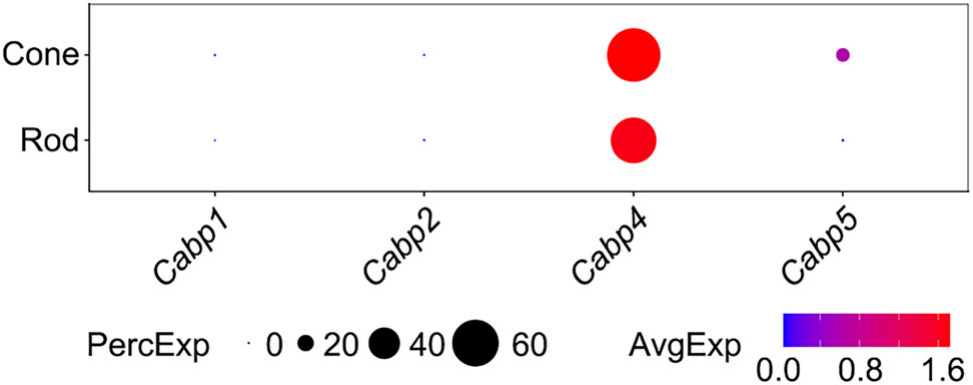
Gene expression patterns of CaBPs in mouse cone and rod photoreceptors. ScRNA-seq analysis reveals that both cone and rod photoreceptors exhibit high levels of CaBP4 transcripts. Note that the *Cabp5* gene, encoding CaBP5, is expressed at a detectable level in cones but is absent in rods. The dot size represents the percentage of cells expressing a specific gene within each cluster (PercExp). The color indicates the average expression level of a gene in those expressing cells (AvgExp). CaBPs, Ca^2+^-binding proteins.

To validate our unexpected findings regarding the complex gene expression patterns in mouse BCs, we conducted a control analysis using the abovementioned scRNA-seq dataset (GEO accession number, GSE63473), which also contains single-cell transcriptomes for mouse cone and rod photoreceptors (Macosko et al., 2015). Consistent with previous reports, both cone and rod photoreceptors expressed high levels of CaBP4 transcripts (Fig. 3; Haeseleer et al., 2004; Shaltiel et al., 2012). Interestingly, we also observed expression of the *Cabp5* gene at a detectable level in cones, but not in rods (Fig. 3), indicative of coexpression of CaBP4 and CaBP5 in at least some mouse cone photoreceptors.

These results therefore demonstrated that most BC types in the mouse retina express distinct combinations of CaBP1, CaBP2, and CaBP5, rather than a single CaBP as commonly assumed. Furthermore, some mouse cone photoreceptors, but not rod photoreceptors, likely coexpress CaBP4 and CaBP5.

### Coexpression of CaBP1 and CaBP5 in mouse RBs is confirmed by scRT-PCR analysis

Our scRNA-seq analysis revealed that mouse RBs coexpressed CaBP1 and CaBP5 (Fig. 2). To further investigate the potential heterogeneity of CaBP1 and CaBP5 expression in individual cells, we undertook a targeted examination of CaBP1 and CaBP5 transcripts in mouse RBs by scRT-PCR. Given that the existing scRNA-seq dataset (GEO accession number, GSE81905) was derived from mouse retinas at P17 (Shekhar et al., 2016), we conducted scRT-PCR experiments using individual RBs from P17 mice as well as from adult mice aged between 6 and 10 weeks. This approach allowed us to investigate whether there might be any developmental changes in the expression of CaBP1 and CaBP5 transcripts.

**Figure 4.**
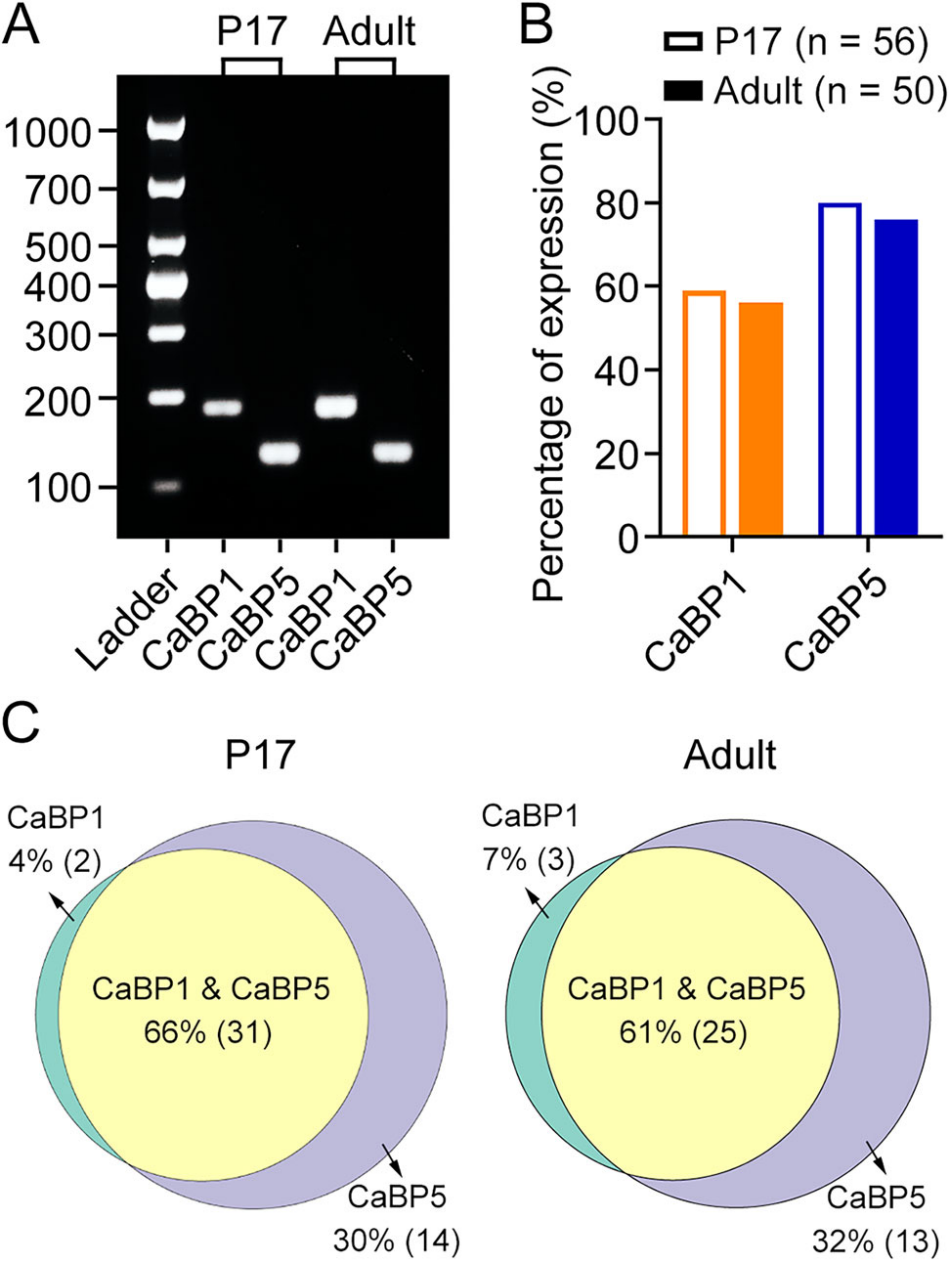
Coexistence of CaBP1 and CaBP5 in mouse RB cells. ***A***, Single-cell RT-PCR analysis reveals coexpression of CaBP1 and CaBP5 in RBs from both P17 and adult mice. A DNA ladder, ranging from 100 to 1,000 bp, is shown for reference on the left. RB, rod bipolar cell; P17, postnatal day 17. ***B***, The percentages of CaBP1 and CaBP5 expression in RBs from both P17 and adult mice (Table 2). ***C***, Schematic diagrams showing the molecular heterogeneity for CaBP1 and CaBP5 expression in individual RBs from both P17 and adult mice (Table 3). The exact number of cells exhibiting each expression pattern is indicated in parentheses.

Consistent with our scRNA-seq analysis (Fig. 2), scRT-PCR detected the presence of CaBP1 and CaBP5 transcripts in individual RBs from both adult and P17 mice (*n* = 50 RBs from adult animals; *n* = 56 RBs from P17 animals; Fig. 4*A,B*; Table 2). Generally, the mRNA expression levels of CaBP1 and CaBP5, as detected by scRT-PCR, were quite stable across the developmental stages, from P17 to adulthood (Fig. 4*B*).

**Table 2.** Expression of CaBP1 and CaBP5 in P17 and adult mouse retinal RBs

	CaBP1	CaBP5
P17 mice		
Total RB number	56	56
Positive RB number	33	45
Negative RB number	23	11
Percentage of expression (%)	58.93	80.36
Adult mice		
Total RB number	50	50
Positive RB number	28	38
Negative RB number	22	12
Percentage of expression (%)	56.00	76.00

CaBP1, Ca^2+^-binding protein 1; CaBP5, Ca^2+^-binding protein 5; P17, postnatal day 17; RB, rod bipolar cell.

It is noteworthy that the expression profiles of CaBP1 and CaBP5 transcripts varied between individual cells, with most RBs coexpressing CaBP1 and CaBP5 (Fig. 4*C*; Table 3). In both P17 and adult mouse retinas, ∼66% (*n* = 31) and 61% (*n* = 25) of RBs expressing at least one of CaBP1 and CaBP5 (*n* = 47 RBs for P17 mice; *n* = 41 RBs for adult mice; Fig. 4*C*; Table 3) displayed this specific combination of transcripts, respectively. This observation indicates that the coexpression of CaBP1 and CaBP5 is a prevalent feature in the vast majority of RBs within the mouse retina.

**Table 3.** Molecular heterogeneity of CaBP1 and CaBP5 expression in individual mouse RBs

	P17 mice		Adult mice	
	RB number	Percentage (%)	RB number	Percentage (%)
CaBP1 alone	2	4.26	3	7.32
CaBP5 alone	14	29.79	13	31.71
CaBP1 and CaBP5	31	65.96	25	60.98
Total	47		41	

CaBP1, Ca^2+^-binding protein 1; CaBP5, Ca^2+^-binding protein 5; P17, postnatal day 17; RB, rod bipolar cell.

### All the three known splice variants of CaBP1 are detected in mouse RBs

To date, three splice variants of CaBP1, including S-CaBP1 (the short form of CaBP1), L-CaBP1 (the long form of CaBP1), and caldendrin, have been characterized (Seidenbecher et al., 1998; Haeseleer et al., 2000; Sokal et al., 2000; Haeseleer and Palczewski, 2002). To identify the specific splice variant(s) expressed in mouse RBs, we performed targeted scRT-PCR experiments using individual CaBP1-expressing RBs with primer pairs specific for each CaBP1 splice variant. Furthermore, we aimed to assess whether there were any developmental changes in the mRNA expression of CaBP1 splice variants by examining RBs from both P17 and adult mice.

**Figure 5.**
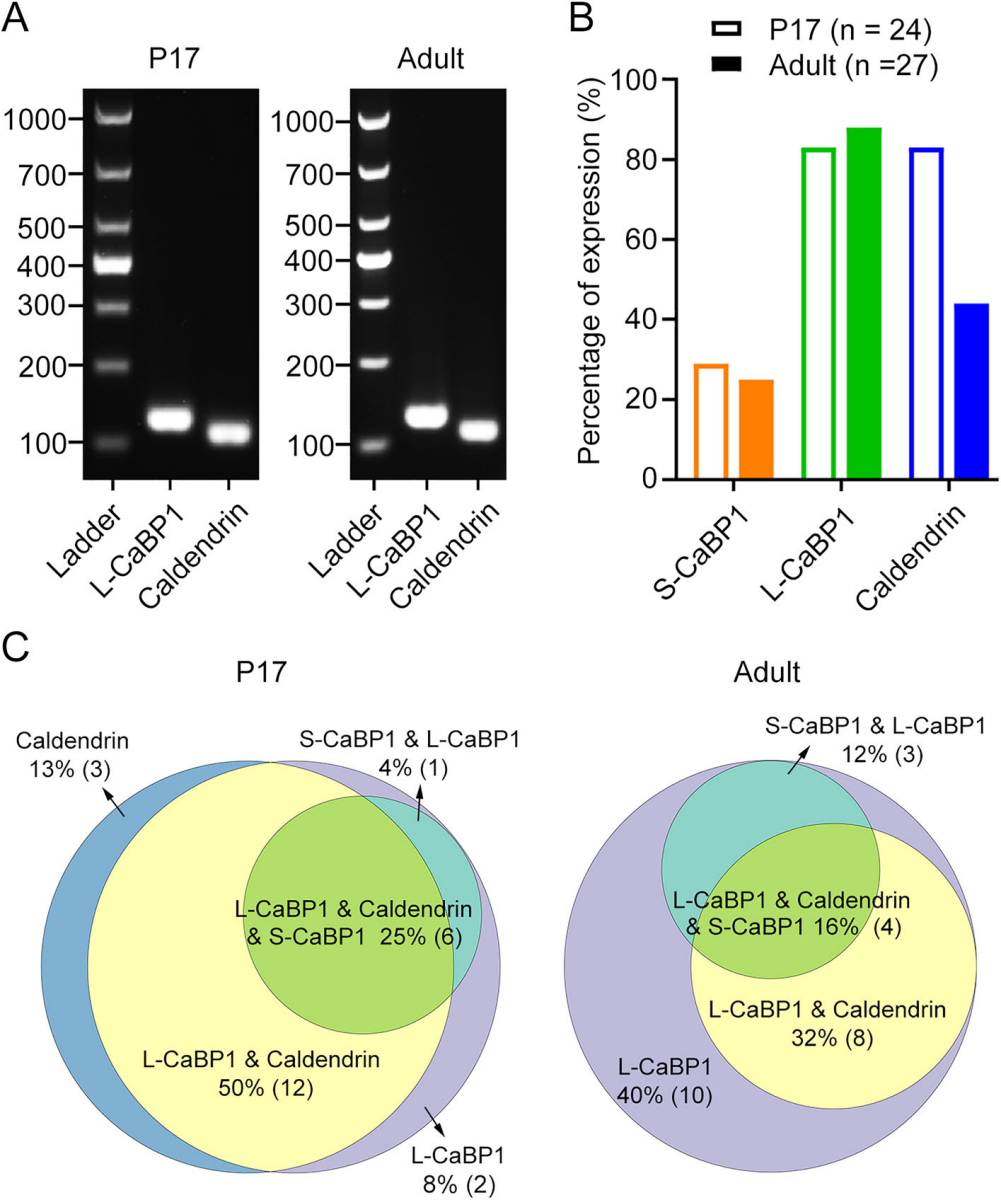
Three splice variants of CaBP1 are detected in mouse RB cells. ***A***, ScRT-PCR analysis reveals coexpression of the long form of CaBP1 (L-CaBP1) and caldendrin in RBs from both P17 (left panel) and adult (right panel) mice. The short form of CaBP1 (S-CaBP1) is also expressed in some RBs (data not shown). A DNA ladder, ranging from 100 to 1,000 bp, is shown for reference on the left. RB, rod bipolar cell; P17, postnatal day 17. ***B***, The percentages of S-CaBP1, L-CaBP1, and caldendrin expression in RBs from both P17 and adult mice (Table 4). Notably, the percentage of caldendrin expression is significantly lower in adult RBs compared with P17 RBs. ***C***, Schematic diagrams showing the high molecular heterogeneity for S-CaBP1, L-CaBP1, and caldendrin expression in individual RBs from both P17 and adult mice (Table 5). The percentages of distinct combinations of S-CaBP1, L-CaBP1, and caldendrin are shown, and the exact number of cells exhibiting each expression pattern is indicated in parentheses.

The mRNAs encoding all three splice variants of CaBP1 were detected in mouse RBs from both P17 and adult mice by scRT-PCR, albeit with significantly different frequencies: mRNAs encoding L-CaBP1 and caldendrin were detected much more frequently than those encoding S-CaBP1 (Fig. 5*A*,*B*; Table 4). While the mRNA expression levels of L-CaBP1 and S-CaBP1 were relatively stable from P17 to adulthood, there was a significant decrease in the expression of caldendrin mRNAs in RBs from the adult mice compared with the P17 stage (Fig. 5*B*; Table 4), suggesting the potential role of caldendrin in the development of mouse retinal RBs.

**Table 4.** Expression of CaBP1 splice variants in P17 and adult mouse retinal RBs

	S-CaBP1	L-CaBP1	caldendrin
P17 mice			
Total RB number	24	24	24
Positive RB number	7	20	20
Negative RB number	17	4	4
Percentage of expression (%)	29.17	83.33	83.33
Adult mice			
Total RB number	27	27	27
Positive RB number	7	24	12
Negative RB number	20	3	15
Percentage of expression (%)	25.93	88.89	44.44

CaBP1, Ca^2+^-binding protein 1; S-CaBP1, the short form of CaBP1; L-CaBP1, the long form of CaBP1; P17, postnatal day 17; RB, rod bipolar cell.

A high degree of heterogeneity in mRNA expression of three CaBP1 splice variants was observed within the population of RBs (Fig. 5*C*; Table 5). In P17 mice, ∼75% of CaBP1-expressing RBs coexpressed L-CaBP1 and caldendrin transcripts (Fig. 5*C*; Table 5). However, in adult mice, the lower percentage (∼48%) of RBs exhibited coexpression of L-CaBP1 and caldendrin mRNAs, and a higher proportion (40%; compared with 8% in P17 mice) of RBs exclusively expressed L-CaBP1 mRNAs (Fig. 5*C*; Table 5), most likely owing to the significant decrease of caldendrin expression observed in adult RBs (Fig. 5*B*; Table 4).

**Table 5.** Molecular heterogeneity of CaBP1 splice variant expression in individual mouse RBs

	P17 mice		Adult mice	
	RB number	Percentage (%)	RB number	Percentage (%)
L-CaBP1 alone	2	8.33	10	40.00
Caldendrin alone	3	12.50	0	0.00
S-CaBP1 and L-CaBP1	1	4.17	3	12.00
L-CaBP1 and caldendrin	12	50.00	8	32.00
S-CaBP1 and L-CaBP1 and caldendrin	6	25.00	4	16.00
Total	24		25	

CaBP1, Ca^2+^-binding protein 1; S-CaBP1, the short form of CaBP1; L-CaBP1, the long form of CaBP1; P17, postnatal day 17; RB, rod bipolar cell.

These results therefore demonstrated that all three CaBP1 splice variants, predominantly L-CaBP1, are detected in mouse retinal RBs, but only the caldendrin expression in RBs undergoes a significant reduction during retinal development.

### The overall expression patterns of CaBPs in mouse and human retinal neurons are similar

To compare the expression patterns of CaBPs in the retinas of mice and humans, we analyzed two existing scRNA-seq datasets (EGA accession number, EGAS00001004561; GEO accession number, GSE148077) that contain transcriptomic profiles of various cell types from both the foveal and peripheral regions of the human retina (Cowan et al., 2020; Yan et al., 2020b). Generally, our analysis of these two datasets revealed comparable expression patterns of CaBPs in human retinal neurons. Hence, for simplicity, we present the results from one of these datasets (EGA accession number, EGAS00001004561) here.

**Figure 6.**
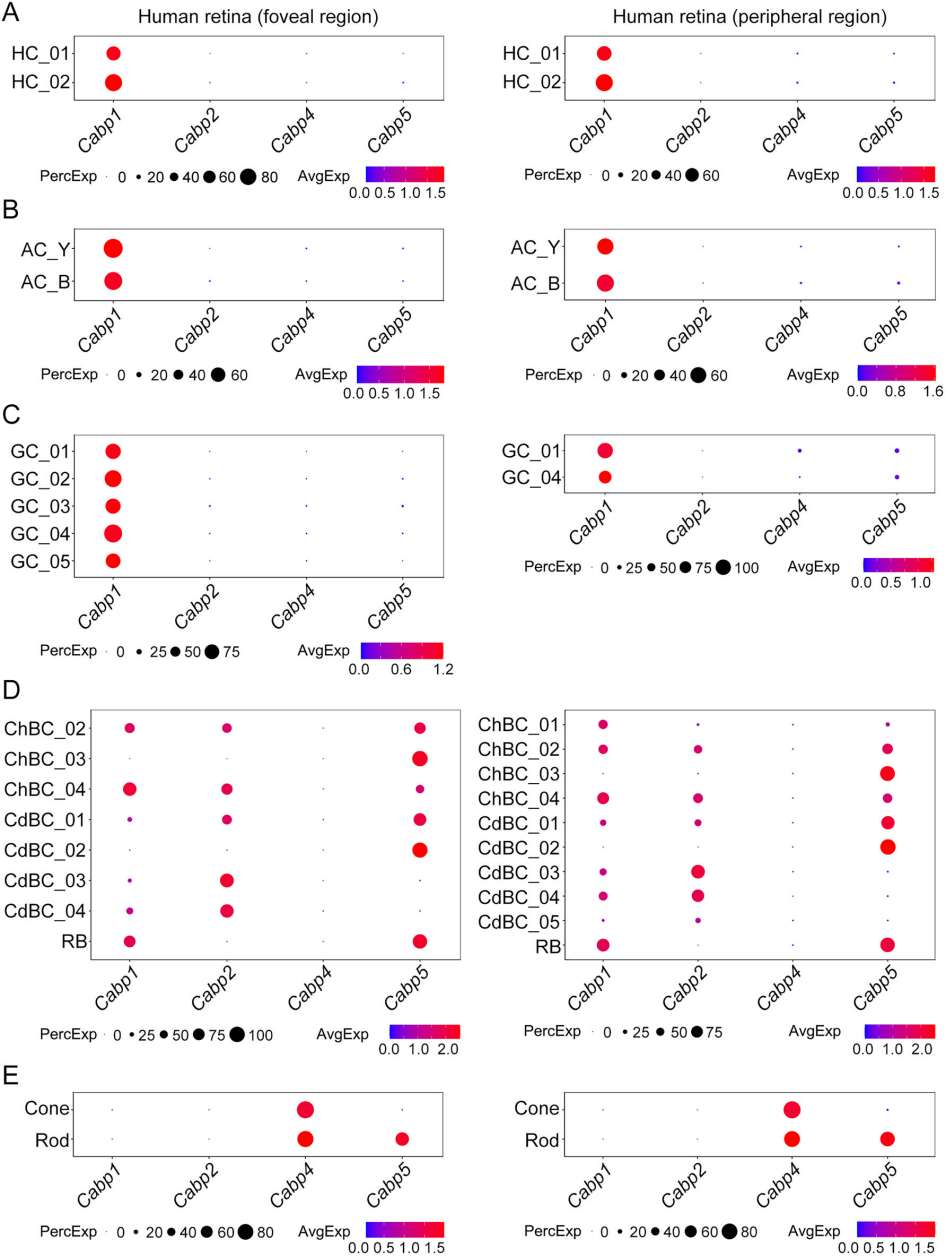
The expression patterns of CaBPs in various cell types of the human retina from the foveal and peripheral regions. scRNA-seq analysis reveals the gene expression patterns of CaBPs in human retinal HCs (***A***), ACs (***B***), GCs (***C***), BCs (***D***), and photoreceptors (***E***). ***A***, Both types of HCs exhibit high-level expression of the *Cabp1* gene encoding CaBP1. ***B***, Both glycinergic and GABAergic ACs (AC_Y and AC_B, respectively) display robust expression of the *Cabp1* gene. ***C***, All GC types, including five types from the foveal region and two types from the peripheral region, exhibit high levels of CaBP1 transcripts. ***D***, Most molecularly defined BC types express distinct combinations of CaBP1, CaBP2, and CaBP5 transcripts. Note that CaBP1 and CaBP5 are coexpressed in RBs. ***E***, Both cone and rod photoreceptors exhibit high levels of CaBP4 transcripts. Notably, a very high level of mRNAs encoding CaBP5 is detected in rod photoreceptors. The dot size represents the percentage of cells expressing a specific gene within each cluster (PercExp). The color indicates the average expression level of a gene in those expressing cells (AvgExp). CaBPs, Ca^2+^-binding proteins; HC, horizontal cell; AC, amacrine cell; GC, ganglion cell; BC, bipolar cell; RB, rod bipolar cell; ChBC, OFF (hyperpolarizing) cone bipolar cell; CdBC, ON (depolarizing) cone bipolar cell.

Despite the discrepancies in GC and BC types, our scRNA-seq analysis did not detect significant differences in the expression patterns of CaBPs between the foveal and peripheral regions of the human retina (Fig. 6; compare left and right panels). Both molecularly defined types of human HCs expressed high levels of CaBP1 transcripts (Fig. 6*A*). Similarly, both glycinergic and GABAergic ACs exclusively expressed CaBP1 transcripts (Fig. 6*B*), and all GC types exhibited high levels of mRNAs encoding CaBP1 (Fig. 6*C*). The gene expression patterns of CaBPs in human retinal BCs were more complex than expected, with most BC types expressing distinct combinations of CaBP1, CaBP2, and CaBP5 transcripts; it's worth noting that coexpression of CaBP1 and CaBP5 transcripts was also observed in human RBs (Fig. 6*D*). Human cone and rod photoreceptors exhibited robust CaBP4 mRNA expression as expected, but contrary to our observations in mice (Fig. 3), human rods, rather than cones, expressed CaBP5 mRNAs at a very high level (Fig. 6*E*).

**Figure 7.**
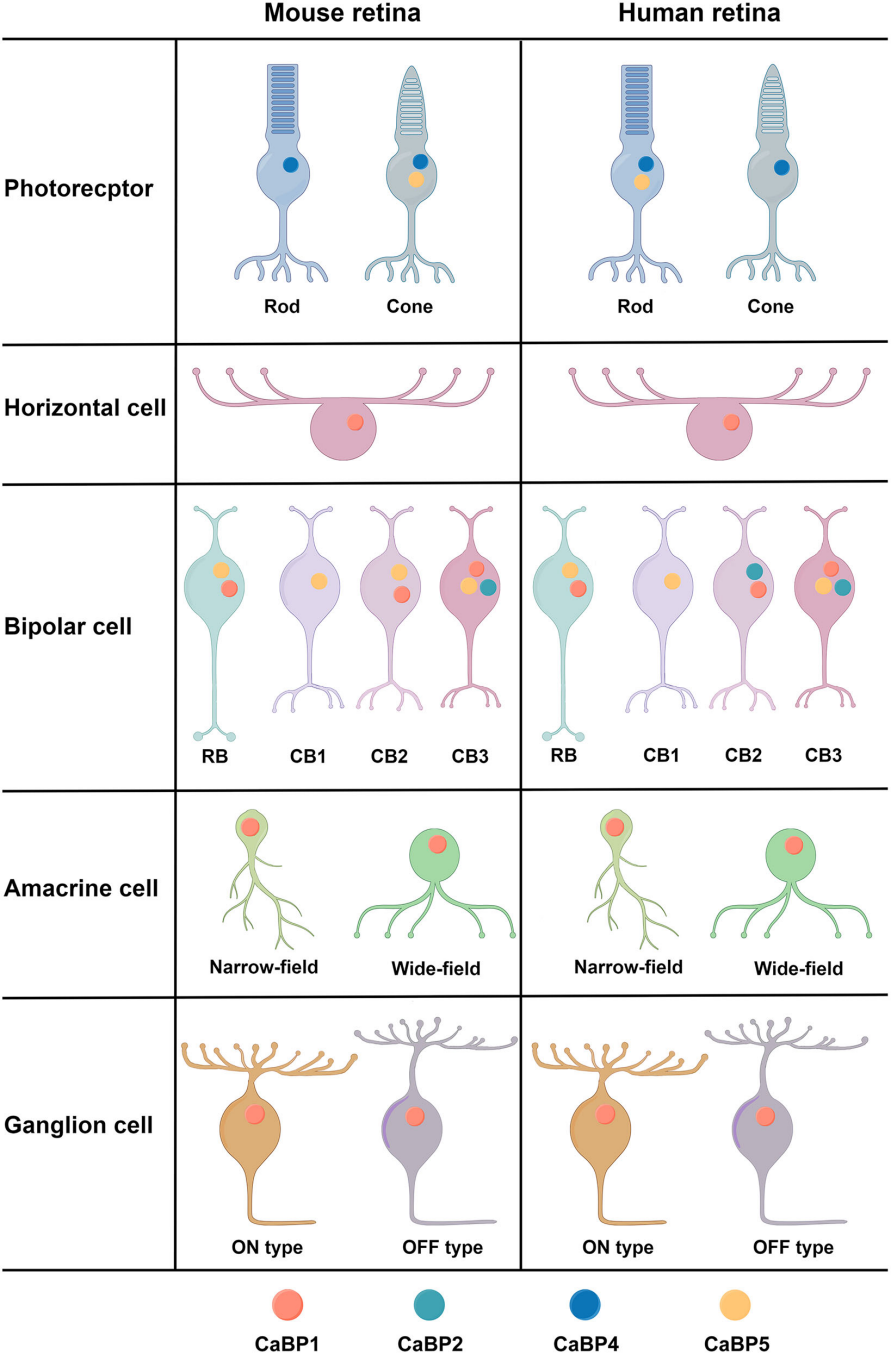
A schematic diagram showing unique expression patterns of CaBPs in mouse and human retinal neurons. While mouse rods and human cones exclusively express CaBP4, mouse cones and human rods coexpress both CaBP4 and CaBP5. Nearly all HC, AC, and GC types exclusively express CaBP1, whereas most bipolar cell types, including RB cells, express different combinations of CaBP1, CaBP2, and CaBP5. Notably, cone bipolar (CB) cells in both mouse and human exhibit three distinct expression patterns (designated as CB1, CB2, and CB3) for CaBP1, CaBP2, and CaBP5.

In summary, our analysis revealed remarkably similar expression patterns of CaBPs in mouse and human retinal neurons; however, while mouse rods and human cones exclusively express CaBP4, mouse cones and human rods coexpress CaBP4 and CaBP5. The unique expression patterns of CaBPs in mouse and human retinal neurons are summarized in a schematic diagram shown in Figure 7.

## Discussion

We combined scRNA-seq and scRT-PCR analyses to provide a comprehensive characterization of CaBPs in mouse and human retinal neurons. Our findings were intriguing: the overall expression patterns of CaBPs in mouse and human retinas exhibited striking similarities. Our work revealed unexpectedly complex expression patterns of CaBPs in both mouse and human BCs—i.e., different combinations of CaBP1, CaBP2, and CaBP5 rather than a single CaBP as previously thought. Furthermore, we demonstrated that CaBP1 and CaBP5 coexisted in mouse RBs. Additionally, we found that both mouse cones and human rods concurrently expressed CaBP4 and CaBP5. Our present study, for the first time, provided direct evidence for the coexpression of multiple CaBPs within individual cells. These findings offer valuable insights into previously puzzling observations from loss-of-function studies involving single CaBP knock-out (KO) animals and patients with CaBP4-related visual disorders, which are not yet fully understood. Finally, as scRNA-seq analysis accurately depicts the expression patterns of CaBPs in RBs, our work can serve as a guide for future studies of the expression and functions of CaBPs in other retinal cell types.

### Coexpression of multiple CaBPs in BCs

Previous studies have reported the distribution of CaBP1, CaBP2, CaBP4, and CaBP5 in the retina, primarily by immunohistochemistry (Menger et al., 1999; Haeseleer et al., 2000; Haverkamp et al., 2003a,b; Schultz et al., 2004; Rieke et al., 2008; Sinha et al., 2016; Pangrsic et al., 2018). CaBP1, CaBP2, and CaBP5 have all been found in some BC types of the mouse retina. CaBP5 is predominantly expressed in RBs, Type 3 OFF cone BCs, and Type 5 ON cone BCs; CaBP1 is found in Types 1 and 2 OFF cone BCs, whereas CaBP2 is present in Type 1 OFF cone BCs and Type 6 ON cone BCs (Haeseleer et al., 2000; Haverkamp et al., 2003a; Rieke et al., 2008; Sinha et al., 2016). It would, then, suggest that CaBP1 and CaBP2 might be coexpressed in Type 1 OFF cone BCs (Sinha et al., 2016), but there is lack of direct evidence (e.g., double labeling experiments) to support this notion. Mice lacking CaBP1, CaBP2, or CaBP5 do not exhibit significant morphological alterations in their retinas or notable differences in b-wave amplitudes of ERGs, which reflect the functional activities of BCs (Rieke et al., 2008; Sinha et al., 2016). Nevertheless, removal of each CaBP leads to altered light responses in GCs, indicating that CaBP1, CaBP2, and CaBP5 play crucial roles in regulating signal transmission within the inner retina (Rieke et al., 2008; Sinha et al., 2016), possibly by affecting neurotransmitter release from BCs (Sokal and Haeseleer, 2011).

In the present work, we found that the expression patterns of CaBPs in retinal BCs were more complex than expected. While almost all AC, GC, and HC types exclusively expressed CaBP1 (Figs. 1, 6), the majority of BC types expressed distinct combinations of CaBP1, CaBP2, and CaBP5 (Figs. 2, 6), rather than just a single CaBP as previously thought. For instance, both mouse and human RBs coexpressed CaBP1 and CaBP5 (Figs. 2, 4, 6). Given the unique and overlapping expression patterns of CaBPs in BCs, the relatively slight changes of gross retinal morphology and ERG recordings in single CaBP KO animals can likely be attributed to functional compensation by other CaBPs. This finding suggests that previous studies of single CaBP KO mice may have been inconclusive in terms of understanding the full functional roles of these proteins. Therefore, to gain a more comprehensive understanding of the functions of CaBP1, CaBP2, and CaBP5 in the retina, future studies employing double or even triple KO animals will be highly beneficial.

The analysis of CaBP1, CaBP2, and CaBP5 expression in BCs using scRNA-seq and scRT-PCR in this study revealed a broader distribution of these proteins in BC types compared with previous immunohistochemical studies, which detected protein expression. This difference is likely due to the higher sensitivity of the scRNA-seq and scRT-PCR methods, which can detect low levels of mRNA expression. However, an alternative explanation could be that high levels of mRNAs do not always translate into corresponding protein levels, possibly due to factors such as alternative splicing or posttranscriptional regulation. The former was more likely to be true since it provided better explanations for previous observations from single CaBP KO animals. Future studies, therefore, will be required to validate the coexpression of multiple CaBPs in individual BCs at the protein level using some other methods such as single-cell proteomics (Budnik et al., 2018; Cheung et al., 2021) or single-cell Western blotting (Hughes et al., 2014).

### Coexpression of CaBP4 and CaBP5 in specific photoreceptor types

It has been well established that CaBP4, which is exclusively expressed at photoreceptor terminals, plays an essential role in retinal functions by regulating the activities of Ca_V_1.4 L-type Ca^2+^ channels (Haeseleer et al., 2004; Shaltiel et al., 2012). Studies in mice with mutations in the *Cabp4* gene have revealed significant visual impairments, with phenotypes resembling those seen in patients with incomplete congenital stationary night blindness (CSNB2; Haeseleer et al., 2004; Zeitz et al., 2006). Indeed, rod functions are severely compromised in CaBP4 KO mice; cone functions, however, appear to be less affected by the removal of CaBP4 (Haeseleer et al., 2004). Surprisingly, human patients with mutations in the *Cabp4* gene present with a different phenotype. Instead of the rod-dominant dysfunction observed in mice, these patients suffer from a condition known as congenital cone-rod synaptic disorder (Littink et al., 2009). Notably, these patients do not report night blindness, a hallmark symptom of CSNB2 (Littink et al., 2009). ERG testing in these patients indicates that cone functions are severely compromised, while rod functions are only mildly affected (Littink et al., 2009; Khan et al., 2013; Khan, 2014).

The apparent contrast between observations in animal models and patients with *Cabp4* gene mutations remains an intriguing puzzle. To date, the expression of CaBPs, apart from CaBP4, in retinal photoreceptors has not been reported. Therefore, we were surprised that our scRNA-seq analysis revealed the coexpression of CaBP4 and CaBP5 in some mouse cones (Fig. 3) and human rods (Fig. 6). In contrast, mouse rods and human cones exclusively expressed CaBP4 (Figs. 3, 6). Consequently, functional compensation by CaBP5 upon removal of CaBP4 likely would help maintain normal or subnormal functions of mouse cones and human rods. The difference in expression patterns of CaBP5 between mouse and human retinal photoreceptors may be attributed to the living habits of these two species: mice are nocturnal animals, while humans are diurnal. However, this hypothesis still requires verification through the collection of data on the expression of CaBP5 and CaBP4 in retinal rods and cones of more species.

According to previous reports, all of CaBP1, CaBP2, CaBP4, and CaBP5 are present in the cochlea (P. S. Yang et al., 2006; Cui et al., 2007; Hardie and Lee, 2016; T. Yang et al., 2016; Picher et al., 2017). While it is well established that CaBP2 is essential for hearing (Schrauwen et al., 2012; Picher et al., 2017), potentially overlapping roles of CaBP1 and CaBP2 in cochlear inner hair cells have also been suggested (T. Yang et al., 2016). Hence, the unique expression patterns of different combinations of CaBPs rather than a single CaBP in some specific cell types likely exist in both sensory organs (i.e., retina and cochlea).

### Multiple CaBP1 splice variants in individual RBs

Unlike CaBP2, CaBP4, and CaBP5, which are restricted to the retina and cochlea, CaBP1/caldendrin is widely expressed in multiple brain regions (Haeseleer et al., 2000; Laube et al., 2002; Bernstein et al., 2003; Kim et al., 2014; T. Yang et al., 2016; Konietzny et al., 2022; Lopez et al., 2023, 2024). CaBP1 competes with calmodulin for binding to L- and P/Q-type Ca^2+^ channels, thereby antagonizing the effects of calmodulin on these channels (Lee et al., 2002; Zhou et al., 2004, 2005; P. S. Yang et al., 2006; Cui et al., 2007; Tippens and Lee, 2007; Findeisen and Minor, 2010; Few et al., 2011; Shaltiel et al., 2012; Oz et al., 2013; Hardie and Lee, 2016; Ames, 2021). Alternative splicing of CaBP1 mRNAs gives rise to multiple splice variants: S-CaBP1, L-CaBP1, and caldendrin (Seidenbecher et al., 1998; Haeseleer et al., 2000; Sokal et al., 2000; Haeseleer and Palczewski, 2002). Caldendrin, the largest splice variant of CaBP1, is confined to the somatodendritic compartment of neurons (Seidenbecher et al., 1998), while S-CaBP1 and L-CaBP1, when expressed in transfected cells, show distinct subcellular localization (e.g., on the plasma membrane or within the membranes of the Golgi apparatus), suggesting that they may play different roles within neurons (Haeseleer et al., 2000; Haynes et al., 2004; Kasri et al., 2004). In dendritic spines, caldendrin interacts with A-kinase anchoring proteins and modulates the phosphorylation and dephosphorylation of postsynaptic receptors (Gorny et al., 2012). Moreover, caldendrin regulates nanodomain F-actin dynamics in dendritic spines by interacting with cortactin and myosin V (Mikhaylova et al., 2018; Konietzny et al., 2022). Notably, despite its unique localization and interactions, caldendrin shares a commonality with S-CaBP1 and L-CaBP1 in its ability to directly bind to IP_3_Rs, thereby inhibiting IP_3_R-mediated Ca^2+^ release (J. Yang et al., 2002; Haynes et al., 2004; Kasri et al., 2004).

Whether these three CaBP1 splice variants may coexist in individual cells, however, is uncertain. In the present work, we found that CaBP1 coexisted with previously reported CaBP5 in mouse RBs (Figs. 2, 4); additionally, all three CaBP1 splice variants were detected in RBs by scRT-PCR, and the vast majority of RBs coexpressed L-CaBP1 and caldendrin, especially at the P17 stage (Fig. 5). We also found that mRNAs encoding caldendrin in RBs underwent a significant reduction from P17 to adulthood (Fig. 5). Interestingly, it has been reported that the expression level of caldendrin in mouse cochlea shows a dramatic increase from P7 to P21 (T. Yang et al., 2016). These results indicate that caldendrin may play an important role in neuronal development. Since recent studies have shown that caldendrin directly couples postsynaptic calcium signals to actin remodeling and endoplasmic reticulum stabilization in dendritic spines (Mikhaylova et al., 2018; Konietzny et al., 2022), it will be interesting to explore in the future whether and how caldendrin may influence the dendritic morphology of RBs during development. Moreover, future studies will be required to address the hypothesis that three CaBP1 splice variants play different roles in RBs.

## Synthesis

Reviewing Editor: Selva Baltan, Oregon Health &Science University Department of Surgery Decisions are customarily a result of the Reviewing Editor and the peer reviewers coming together and discussing their recommendations until a consensus is reached. When revisions are invited, a fact-based synthesis statement explaining their decision and outlining what is needed to prepare a revision will be listed below. The following reviewer(s) agreed to reveal their identity: NONE.

Dear authors,

Please diligently attend to the revision points raised. If your revision addresses all points satisfactorily, the reviewers have waived the revised version per my decision.
