# Erratum: “Comprehensive Characterization of a Subfamily of Ca^2+^-Binding Proteins in Mouse and Human Retinal Neurons at Single-Cell Resolution”

**DOI:** 10.1523/ENEURO.0101-25.2025

**Published:** 2025-03-17

In the article “Comprehensive Characterization of a Subfamily of Ca^2+^-Binding Proteins in Mouse and Human Retinal Neurons at Single-Cell Resolution” by Jun-Bin Liu, He-Lan Yuan, Gong Zhang, and Jiang-Bin Ke which was published online on September 11, 2024, there were two typographical errors in the Materials and Methods section. In the descriptions of two existing scRNA-seq datasets (GEO accession numbers: GSE137400 and GSE149715), ACs and GCs were transposed. The first sentence of the “Analysis of scRNA-seq datasets” subsection should read: “…GSE137400 (featuring transcriptomes of GCs derived from P56 mice), and GSE149715 (featuring transcriptomes of ACs derived from P19 mice), were reanalyzed….”

These changes do not affect the conclusion of the paper. The authors regret the error, and the online version has been corrected.

